# Serving the scientific community: *FEBS Open Bio* in 2024

**DOI:** 10.1002/2211-5463.13747

**Published:** 2024-01-02

**Authors:** Duncan E. Wright, Miguel A. De la Rosa

**Affiliations:** ^1^ FEBS Open Bio Editorial Office Cambridge UK

## Abstract

*FEBS Open Bio* is committed to not only publishing sound science but also to supporting early‐career researchers and the scientific community as a whole. In this editorial, we look back at how the journal recognised and rewarded excellent research in 2023 and look forward to 2024.

## Graphical abstract

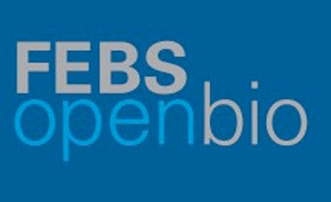

As a not‐for‐profit journal owned by the Federation of European Biochemical Societies (FEBS), *FEBS Open Bio* is committed to not only the rapid publication of sound science but also to supporting research in the molecular life sciences. To this end, the journal both indirectly supports the charitable activities of FEBS through the revenue raised through article publication charges, and also directly organises free webinars, poster and speed talk prizes for early‐career researchers, the annual *FEBS Open Bio* Lecture and the FEBS Congress supplement issue.

In 2023, the journal continued to innovate and improve, and we are looking forward to the developments that 2024 will bring. In this editorial, we look back at the events of 2023 and introduce our plans for the new year ahead.

## Image integrity

Academic research and the life sciences, in particular, are currently facing a serious problem in the form of paper mills, an entire industry devoted to the production of fraudulent research papers in exchange for payment [1]. If this problem were not enough, research continues to be haunted by the twin spectres of individual misconduct and honest mistakes in reporting, with both issues arguably exacerbated by the ease with which researchers can now use image manipulation software to either intentionally manipulate results or generate figures without carefully ensuring that the presented data are correct.

FEBS Press was among the first publishers to employ an in‐house image integrity analyst, Jana Christopher, to help combat fraud and errors in manuscripts submitted to the journal. Through Jana's ongoing hard work, we can detect and reject potential paper mill articles. We have also recently retracted several paper mill articles that originally escaped detection, with the problems brought to light through the use of new software that is able to detect duplications between papers. We strive to stay one step ahead of the paper mills by utilising new tools available to us [2]. For several years, we have sent the figures of articles in provisionally accepted manuscripts for image analysis. In 2023, we decided to begin carrying out image analysis in parallel with peer review of revised manuscripts, thereby bringing academic editors and reviewers into the process. Only through the combined efforts of journals, editors, image analysis experts, software developers, reviewers and authors can we hope to combat the ongoing threat of paper mills.

## In the Limelight issues

In 2023, we were delighted to publish three exciting ‘In the Limelight’ issues, which were focused on (a) RNA biology, (b) the work of FEBS fellows and (c) the involvement of glycosphingolipids in disease. The RNA biology issue, published in June 2023 and edited by guest editor Cecília Maria Arraiano of the ‘Control of Gene Expression’ group at Instituto de Tecnologia Química e Biológica, Universidade NOVA de Lisboa, Oeiras, Portugal, contains three excellent articles focused on different aspects of RNA biology: (i) the role of ribonucleases in disease; (ii) the role of RNA regulators in bacterial biofilm formation; and (iii) the detection of RNA viruses. In the first article, Sandra C. Viegas and colleagues highlight how ribonucleases in both host and pathogen influence disease: mutations in the human Dis3L2 gene are associated with a number of diseases, and bacterial ribonucleases are important for pathogenicity [3]. Bacterial ribonucleases, together with other RNA regulators, are also involved in biofilm formation, as reviewed by Vânia Pobre and colleagues in the second article in the issue [4]. In the final article, So Nakagawa, Takashi Gojobori and colleagues introduce new developments in bioinformatics software and databases for the identification of RNA viruses and discuss viromes from various sources [5]. This issue also features an interview with Cecília, in which she discusses both her research and supporting women in science through her former position as Chair of the FEBS Working Group on Women in Science [6].

Our second ‘In the Limelight’ issue of 2023 took a different approach, focusing not on a specific research area but on the work of FEBS Fellows, representing one of the last cohorts of the Long‐Term Fellowships programme (an initiative that has now been retired and replaced with the new FEBS Excellence Award scheme). This issue was edited by guest editors Alain Krol, director of research emeritus at the CNRS and professor conventionné at the University of Strasbourg, Chair of the FEBS Fellowships Committee, and Miguel De la Rosa, Professor at the University of Seville and Secretary General of FEBS. We are delighted that so many FEBS Fellows contributed Mini‐Reviews or Research Protocols to this special issue and are proud to be able to support the work of early‐career researchers.

Our third In the Limelight issue of 2023 featured eight review articles on the role of glycosphingolipids in human disease, as commissioned by guest editor Sandro Sonnino, Professor in the School of Medicine at the University of Milan. This issue featured eight review articles (the most in any ‘In the Limelight’ issue to date) that cover a broad spectrum of diseases, including Parkinson's disease, infertility, encephalomyeloradiculoneuropathy and parasitic infections. This issue's articles are introduced in depth in an interview with Sandro, in which he also discusses the ongoing importance of research on glycosphingolipids [7].

## The 47th FEBS Congress

The 47th FEBS Congress was held in Tours, France, from 8th to 12th July 2023. This highly successful event marked the beginning of a new era for the annual FEBS Congress, as it was the first to involve the four FEBS Press journals (*The FEBS Journal*, *FEBS Letters*, *FEBS Open Bio* and *Molecular Oncology*) in the organisation of the scientific content. Together with the Société Française de Biochimie et Biologie Moléculaire (SFBBM), the Editors‐in‐Chief of the four journals were involved in selecting the topics and chairs of the seminar sessions. In addition, last year's Congress featured the plenary *FEBS Open Bio* Lecture, which was presented by Jose Rizo‐Rey (Josep Rizo), Professor in the Departments of Biophysics, Biochemistry and Pharmacology of the University of Texas Southwestern Medical Center at Dallas, USA. Jose gave an illuminating, entertaining and thought‐provoking lecture entitled ‘Molecular mechanisms underlying neurotransmitter release and its regulation,’ and received the first *FEBS Open Bio* award. The second *FEBS Open Bio* Lecture will be presented by Sandro Sonnino at the 48th FEBS Congress in Milano, Italy, which will take place from 29th June to 3rd July 2024. We encourage all of our readers to register for this year's congress, which promises to be an excellent and exciting event.

In 2021, *FEBS Open Bio* started to award an annual *FEBS Open Bio* Article Prize to an early‐career researcher who had authored a paper of special interest published in the journal in the previous 18 months. The 2023 prize was awarded to Shinji Sakamoto (formerly of Johns Hopkins University School of Medicine, Baltimore, MD, USA and now affiliated with the Department of Neuropsychiatry, Okayama University Graduate School of Medicine) as the first author of the winning article ‘Alterations in circulating extracellular vesicles underlie social stress‐induced behaviors in mice’ [8]. The winning paper was selected by a jury comprised of three members of the journal's Editorial Board: Alberto Alape Giron (Department of Biochemistry, School of Medicine, Universidad de Costa Rica, San José, Costa Rica), Takashi Gojobori (National Institute of Genetics, Mishima, Shizuoka, Japan) and Cláudio M. Soares (ITQB NOVA, Universidade Nova de Lisboa, Oeiras, Portugal). As part of the prize, Shinji received a registration fee waiver and financial support to cover the costs of travel and accommodation to the FEBS Congress, where he presented an excellent poster on his research. We would like to take this opportunity to congratulate Shinji Sakamoto for winning the prize and wish him ongoing success with his research.

The journal has awarded a poster prize at the FEBS Congress for many years and starting in 2022, began to award poster prizes at other international scientific meetings as well. In 2023, we awarded 31 poster prizes to early‐career researchers at 25 events across 18 countries. In addition, we awarded two speed talk prizes at the 47th FEBS Congress and one oral presentation prize at the Young Scientist's Forum. Details of all poster prizes from 2021 onwards can be viewed on the journal's website here: https://febs.onlinelibrary.wiley.com/hub/journal/22115463/febs‐congress.

*FEBS Open Bio* is proud to be the home for the annual FEBS Congress supplement issue, which contains the abstracts for all talks and posters presented at the event. In 2023, we also published the abstracts for the FEBS‐IUBMB‐ENABLE 1st International Molecular Biosciences PhD and Postdoc Conference, which was held from 16 to 18 November 2022, at the Institute of Biomedicine of Seville, Spain. The meeting was a great success and was followed by a second meeting in Cologne, Germany in November 2023, with a third meeting to be held in Singapore scheduled for 2024. Congress organisers Marta Reyes‐Corral and Maren Pfirrmann co‐authored an in‐depth meeting report for the inaugural meeting, which we invite you to read and hope will inspire you to join the 2024 meeting [9].

Finally, we were also delighted to sponsor a *FEBS Open Bio* Lecture at the Randall Centre for Cell & Molecular Biophysics at King's College London in 2023. The lecture, entitled ‘Linking force, form and function in intestinal organoids and tumoroids’, was presented by Xavier Trepat (ICREA Research Professor, Institute for Bioengineering of Catalonia, Barcelona, Spain) at the centre on 7 June 2023. Ioannis Tsagakis of the journal's editorial office also attended, and this was an excellent opportunity for the journal to connect with our readers.

## Developments in 2023

Following the introduction of our free webinar series in 2022, we held two successful webinars in 2023. The first of these webinars, ‘How much can we trust crystal and NMR structures of weak protein complexes? Lessons from studies of neurotransmitter release’, was presented by Jose Rizo (UT Southwestern Medical Center, Dallas, Texas, USA) on 17 February 2023, and accompanied our special issue on the same topic published in November 2022. In this webinar, Jose Rizo discussed the limitations of crystal and NMR structures when investigating weak protein interactions, using examples from the study of neurotransmitter release.

Our second webinar in 2023, held on 11 October, was arranged to tie in with the ‘In the Limelight’ issue on the work of FEBS fellows. Alain Krol (Chair of the FEBS Fellowships Committee, CNRS and University of Strasbourg) discussed how FEBS can help early‐career researchers through the FEBS Excellence Awards and FEBS fellowships and how to successfully apply for grants and publish research in high‐quality journals. Alain was joined by three early‐career researchers and FEBS Fellows: Geula Hanin (The University of Cambridge), Yan Ma (University of Lausanne) and Pierre Santucci (The Francis Crick Institute), who shared their own experiences. Both webinars were well attended, and we invite you to watch recordings of both webinars online here: https://febs.onlinelibrary.wiley.com/journal/22115463/webinar.

Our top priority for *FEBS Open Bio* is to ensure fast, fair and thorough peer review. To help us maintain this standard, we are constantly looking for ways to better facilitate the peer review process. In 2023, we set up a new Peer Review Task Force, composed of researchers who have agreed to help review manuscripts for which we are struggling to find willing reviewers. The members of the task force, recruited from our Editorial Advisory Board (EAB), are all strongly aligned with our commitment to ensuring efficient peer review. We would like to offer our sincere thanks to the new members of the Peer Review Task Force, who are shown below:

**Jonathan Martinez‐Fabregas**
CABIMER, Universidad de Sevilla‐CSIC, Seville, Spain
**Indira D. Pokkunuri**
University of Houston, USA
**Heinz‐Peter Nasheuer**
Centre for Chromosome Biology & Biochemistry, School of Natural Sciences, NUI Galway, Galway, Ireland
**Tsukasa Tominari**
National Institute of Neuroscience, National Center of Neurology and Psychiatry, Tokyo, Japan

We were also delighted to welcome 11 new members of the EAB to the journal in 2023. The EAB is also committed to helping us with peer review, and their efforts are instrumental in the success of the journal:

**Ammar AL‐Farga**
Department of Biochemistry, College of Science University of Jeddah, Saudi Arabia
**Subashchandrabose Chinnathambi**
National Institute of Mental Health and Neuro Sciences (NIMHANS), Bangalore, India
**Luis Izquierdo**
Barcelona Institute for Global Health (ISGlobal), Hospital Clínic—University of Barcelona, Barcelona, Spain
**Bilon Khambu**
Liver Pathobiology Lab, Department of Pathology and Laboratory Medicine, Tulane University School of Medicine, New Orleans, Louisiana
**Pawel Kozielewicz**
Department of Physiology and Pharmacology, Karolinska Institute, Solna, Sweden
**Petar Ozretić**
Division of Molecular Medicine, Ruđer Bošković Institute, Zagreb, Croatia
**Fabien Pierrel**
French National Centre for Scientific Research (CNRS), TIMC laboratory, Grenoble‐Alpes University, France
**Rituraj Purohit**
CSIR‐Institute of Himalayan Bioresource Technology (CSIR‐IHBT), Palampur, India
**Alexis “Stocko” Stamatikos**
Clemson University, Clemson, SC, USA
**Beimeng Yang**
Roche Innovation Center, Shanghai, China
**Shi‐Bing Yang**
Institute of Biomedical Sciences, Academia Sinica, Taiwan

## Looking ahead

The next 12 months look set to be another exciting year for *FEBS Open Bio*. Our first ‘In the Limelight’ issue of the year, which is focused on Alzheimer's disease and edited by guest editor Koji Yamanaka (Nagoya University, Japan), is set to be published next month. Future special issues in 2024 will be centred on mitochondrial biology and bioinformatics, with other issues planned for publication later in the year. We are also excited to announce that we will be hosting a webinar on the role of glycosphingolipids in disease on 21st February at 16:00 CET. This webinar, which will be presented by Sandro Sonnino (University of Milan, Italy), has been arranged to accompany our recent special issue on the same topic. For details on how to register for the webinar, please keep an eye on our website here: https://febs.onlinelibrary.wiley.com/journal/22115463/webinar.

*FEBS Open Bio* is distinguished from the three other FEBS Press journals (*The FEBS Journal*, *FEBS Letters* and *Molecular Oncology*) by its editorial policy, which is primarily based on the soundness of science: ‘the journal's rigorous peer review process focusses on the technical and ethical quality of papers, rather than subjective judgements of significance’. We are currently in the process of extending our view of the journal to ‘a useful platform for scientists to publish their research work and experimental findings in a high‐quality journal’, and look forward to providing more information on this in 2024.

As ever, we would like to extend our gratitude to all of our editors, authors, colleagues, readers and reviewers for their ongoing support. We look forward to working with all of you in 2024!

## Conflict of interest

The authors declare no conflict of interest.

## Author contributions

DEW and MAR wrote the editorial.
